# Minimally invasive and computer-navigated total hip arthroplasty: a qualitative and systematic review of the literature

**DOI:** 10.1186/1471-2474-11-92

**Published:** 2010-05-17

**Authors:** Inge HF Reininga, Wiebren Zijlstra, Robert Wagenmakers, Alexander L Boerboom, Bregtje P Huijbers, Johan W Groothoff, Sjoerd K Bulstra, Martin Stevens

**Affiliations:** 1 Department of Orthopaedics, University Medical Center Groningen, University of Groningen, The Netherlands; 2 Center for Human Movement Sciences, University Medical Center Groningen, University of Groningen, The Netherlands; 3 Department of Health Sciences, University Medical Center Groningen, University of Groningen, The Netherlands

## Abstract

**Background:**

Both minimally invasive surgery (MIS) and computer-assisted surgery (CAS) for total hip arthroplasty (THA) have gained popularity in recent years. We conducted a qualitative and systematic review to assess the effectiveness of MIS, CAS and computer-assisted MIS for THA.

**Methods:**

An extensive computerised literature search of PubMed, Medline, Embase and OVIDSP was conducted. Both randomised clinical trials and controlled clinical trials on the effectiveness of MIS, CAS and computer-assisted MIS for THA were included. Methodological quality was independently assessed by two reviewers. Effect estimates were calculated and a best-evidence synthesis was performed.

**Results:**

Four high-quality and 14 medium-quality studies with MIS THA as study contrast, and three high-quality and four medium-quality studies with CAS THA as study contrast were included. No studies with computer-assisted MIS for THA as study contrast were identified. Strong evidence was found for a decrease in operative time and intraoperative blood loss for MIS THA, with no difference in complication rates and risk for acetabular outliers. Strong evidence exists that there is no difference in physical functioning, measured either by questionnaires or by gait analysis. Moderate evidence was found for a shorter length of hospital stay after MIS THA. Conflicting evidence was found for a positive effect of MIS THA on pain in the early postoperative period, but that effect diminished after three months postoperatively. Strong evidence was found for an increase in operative time for CAS THA, and limited evidence was found for a decrease in intraoperative blood loss. Furthermore, strong evidence was found for no difference in complication rates, as well as for a significantly lower risk for acetabular outliers.

**Conclusions:**

The results indicate that MIS THA is a safe surgical procedure, without increases in operative time, blood loss, operative complication rates and component malposition rates. However, the beneficial effect of MIS THA on functional recovery has to be proven. The results also indicate that CAS THA, though resulting in an increase in operative time, may have a positive effect on operative blood loss and operative complication rates. More importantly, the use of CAS results in better positioning of acetabular component of the prosthesis.

## Background

Total hip arthroplasty (THA) is considered to be one of the most successful orthopaedic interventions of the past 40 years, with 10-year survival rates exceeding 90% [1,2]. In recent decades there has been considerable effort to improve the component designs and modes of fixation of total hip prostheses [3]. The concept of minimally invasive surgery (MIS) was adopted recently in the orthopaedic society, leading to the development of minimally invasive techniques for THA. Computer-assisted surgery (CAS) has also gained popularity, since it has the potential to improve the accuracy of orthopaedic procedures.

Despite the increase in use of MIS THA, its risks and benefits are still an ongoing debate issue in the orthopaedic society. Proponents of MIS THA claim that it results in less soft-tissue trauma (smaller skin incision and less muscle damage), reduced blood loss and fewer blood transfusion requirements. Postoperative benefits include less pain, shorter hospital stay, quicker return to function and better cosmetic appearance [4,5]. Opponents claim that MIS THA introduces additional risks due to limited visibility of anatomical landmarks and vital structures [6]. Complications involve higher risks for thromboembolism, infection, neurovascular injury, femoral fracture and component malposition, which can result in increased prosthetic wear [7,8].

Proper positioning of the hip prosthesis is essential for improving the long-term success of THA. Higher rates of pelvic osteolysis, asymmetric polyethylene wear and component migration have been observed when the acetabular component is malpositioned [9]. Lewinnek et al. [10] determined a "safe zone" of 5° to 25° of anteversion and 30° to 50° of abduction. They found that the dislocation rate of hip prostheses, where the acetabular components were placed outside this safe range, was approximately four times higher. Most surgeons aim for this safe zone using mechanical alignment guides provided by the manufacturer of the hip prosthesis. However, these mechanical alignment guides have shown clear limitations in terms of accuracy and precision of proper orientation of the hip prosthesis [11].

As a result, the interest in computer navigation systems for orientation of the hip prosthesis is increasing, since it may be the solution for the aforementioned problems related to proper prosthetic positioning. Moreover, CAS is not only aimed at an improved alignment of the hip prosthesis, it also provides instant information and feedback to the surgeon, which may make the surgical technique easier to perform and may result in better clinical outcomes. The imaging systems that are used during CAS can be roughly divided into image-based and imageless systems. Image-based systems require the collection of morphological information by preoperative CT scans or MRI, or by means of intraoperative fluoroscopy. Imageless systems use a virtual anatomical model which is embedded in the software and is supplemented by intraoperative registration data of anatomical landmarks [12].

CAS in THA is not very common nowadays, due to the fact that current CAS systems may involve longer operation times and the introduction of new equipment in the operating room. Other factors that limit the broad application of CAS are costs and complexity of computer navigation systems [13]. Several studies have shown however that inaccuracies in prosthetic placement through conventional THA techniques can be significantly reduced by using computer navigation, thereby reducing the risk of various complications such as dislocations [14-16].

The use of CAS may be the solution to the limited visibility of anatomical landmarks during MIS THA [17]. Some even hypothesize that MIS in combination with CAS will result in better positioning of the prosthesis, compared to conventional THA techniques [18]. Combining both techniques with claims of quicker recovery and less pain, together with accurate acetabular component positioning and a minimized risk of dislocation, may result in a more effective procedure for THA compared to the conventional technique. However, there is still controversy concerning the most effective technique for THA because of a lack of scientific evidence on the effectiveness of MIS, CAS and computer-assisted MIS for THA. Hence we performed a systematic review of published evidence on the effectiveness of MIS, CAS and computer-assisted MIS for THA.

## Materials and methods

### Search strategy

Following the recommendations of the Cochrane collaborations, an extensive computerised literature search of PubMed, Medline, Embase and OVIDSP was conducted on all studies published between 1995 and May 2009. We used database-appropriate terms, including hip arthroplasty(ies)/replacement(s), minimally invasive/MIS/mini-incision, and/or computer-assisted/navigation/CAS/CAOS. The search strategy was formulated by an experienced medical librarian. To find more studies, the reference lists of all relevant studies were reviewed for potential articles.

### Inclusion criteria and procedure

A study was included in the review if 1) a randomized controlled trial or a clinical controlled trials was conducted; 2) the study was published in English, Dutch or German; 3) the study was a full-length published article or fully-written published report; 4) the study population comprised patients aged 18 years or older who were undergoing a THA; 5) the study group and control group were similar at baseline with respect to age, gender and BMI; 6) the study contrast was minimally invasive total hip arthroplasty, computer-assisted total hip arthroplasty or a combination of both; and 7) at least one of the following outcome measures was assessed: operative outcome including blood loss and operative time; length of hospital stay; adverse events including intraoperative and postoperative complications; radiographic outcomes including number of outliers of acetabular components outside the desired alignment range; and/or one of the Outcome Measures in Rheumatology Clinical Trials (OMERACT) [19]: pain, self-reported physical function and observed physical function, with a follow-up of at least 6 weeks up to one year postoperatively.

The procedure for inclusion of studies was based on the recommendations described by Van Tulder et al. [20] The study selection was performed in two stages. The first selection, based on titles and abstracts and taking in consideration the inclusion criteria, was independently performed by two reviewers (IHFR and BPH). The next stage in the inclusion procedure was performed by the same two reviewers, who independently applied the selection criteria as stated above using the full reports. Disagreement was resolved by discussion. If agreement was not achieved at any stage, a third reviewer was consulted (WZ).

### Assessment of methodological quality

The methodological quality of all articles was independently assessed by two reviewers (IHFR and BPH) using a criteria list [20]. This list contains 11 criteria related to selection bias, performance bias, attrition bias and detection bias. The requirement of blinding patients or care providers (in this case orthopaedic surgeons) to the intervention (THA) was excluded because such blinding is not possible in this type of research. The question about acceptable compliance in all groups was also excluded, since the question was not applicable to this type of research. All criteria were scored as "yes", "no" or "unclear". Studies were considered to be of methodologically high quality when at least six items scored positively; a score of 3 to 5 was medium quality and a score below 3 was considered low quality. Table 1 shows the used criteria list. Disagreement was resolved by discussion and a third reviewer (WZ) was consulted if disagreement persisted.

**Table 1:** Methodological quality criteria list

Item	Description
1	Was the method of randomization adequate?
2	Was the treatment allocation concealed?
3	Were the groups similar at baseline regarding the most important prognostic indicators?
4	Was the outcome assessor blinded to the intervention?
5	Were co-interventions avoided or similar?
6	Was the drop-out rate described and acceptable?
7	Was the timing of the outcome assessment similar in all groups?
8	Did the analysis include an intention-to-treat analysis?

### Statistical analysis

Analysis of the extracted data from the included articles was conducted in line with guidelines for systematic reviews from the Cochrane Collaboration Back Review Group [20]. For continuous variables, the standardised mean difference (SMD) with corresponding 95% confidence intervals (95% CIs) was calculated whenever possible. These effect estimates were interpreted according to Cohen: an SMD of 0.2-0.4 was considered a small effect, 0.5-0.7 moderate and ≥0.8 large [21]. For dichotomous outcomes such as postoperative complications and acetabular outliers the odds ratio (OR) and 95% CIs were calculated as the summary statistics. This ratio represents the odds of complications or acetabular outliers occurring in the study group compared with the control group. An odds ratio of less than 1 favours the study group and the point estimate of the odds ratio is considered to be statistically significant if the 95% CI does not include the value of 1. Analysis of the included articles was conducted using Review Manager 5 (version 5.0.18, The Nordic Cochrane Centre, The Cochrane Collaboration, Copenhagen, Denmark).

Efforts to retrieve raw data or means and their standard deviations to compute effect sizes or odds ratios by contacting the authors of articles where these data were not reported, were unsuccessful. We therefore chose to summarise the results by means of a qualitative analysis using a rating system that consists of five levels of scientific evidence, taking into account the methodological quality and the outcome of the original studies (best-evidence synthesis) (Table 2) [20].

**Table 2:** Best-evidence synthesis

Strong evidence	Consistent findings among multiple high-quality trials*
Moderate evidence	Consistent findings in multiple low-quality trials and/or one high-quality trial
Limited evidence	Consistent findings in at least one low-quality trial
Conflicting evidence	Inconsistent findings among multiple trials (high- and/or low-quality trials)
No evidence	Findings of eligible trials do not meet the criteria for one of the levels of evidence stated above, or there are no eligible trials available

* Consistent findings were defined as ≥75% of the trials showing results in the same direction.

## Results

### Selection of studies

**Figure 1:**
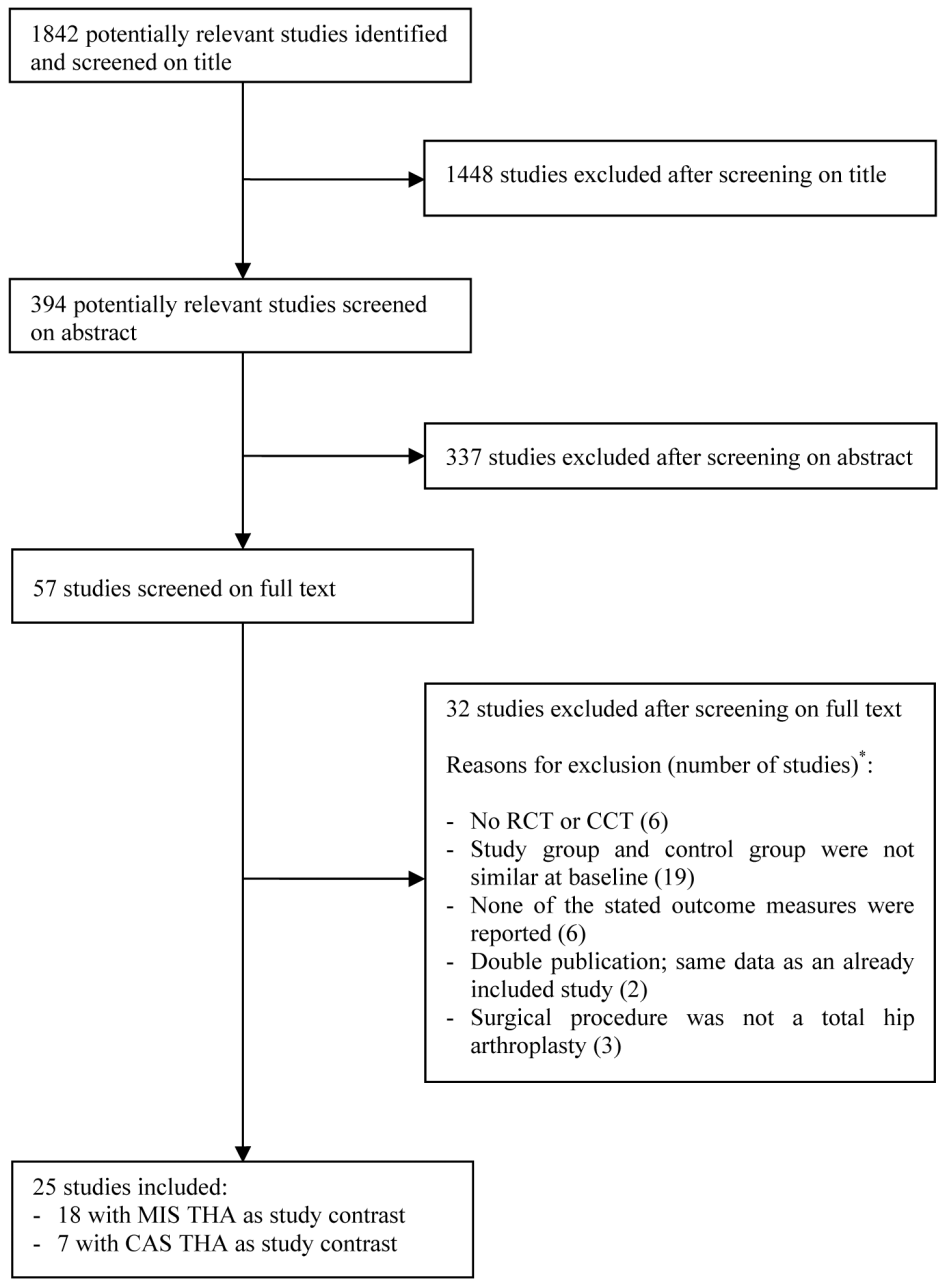
**Flow chart of inclusion procedure**. * Multiple reasons for excluding were possible per study. RCT = randomized controlled trial; CCT = controlled clinical trial; MIS THA = minimally invasive total hip arthroplasty; CAS THA = computer-assisted total hip arthroplasty.

Since the search strategy for MIS, CAS and computer-assisted MIS for THA contained similar components, the results of these search strategies overlapped. After removing double citations, 1841 citations remained. A flow chart of the results of the selection procedure after selection based on title, abstract and full text is shown in Figure 1. The main reasons for exclusion of potentially relevant studies based on full-text articles are also presented in Figure 1.

Eventually, 25 articles were included. In 18 of these articles the study contrast was minimally invasive THA [4,17,22-37]. A computer navigation system was used during THA during the conventional as well as the MIS approach in two of these studies [17,26]. Computer-assisted THA was the study contrast in seven articles [15,16,18,38-41]. In two of these studies, a minimally invasive technique for THA was used in the freehand as well as the CAS group [18,39]. In the study of Kalteis et al. [15], acetabular components were implanted either freehand or using a CT-based or an imageless computer navigation system. The results of the comparison of the two navigation systems are reported separately in this review. Najarian et al. [39] report on the results of the first 49 cases of CAS THA and a second series of 47 cases of CAS THA. Since the first series were used to present data on the learning curve of CAS THA, the results of the second series are reported in this review.

None of the included articles had computer-assisted minimally invasive THA as study contrast. The characteristics of the included studies are presented in Additional file 1.

### Methodological quality

The results of the methodological quality assessment of the included articles are presented in Table 3. Overall, the methodological quality of the studies was found to be medium. Four of the studies with MIS THA as study contrast were of high methodological quality [4,30,31,34] and 14 of medium methodological quality [17,22-29,32,33,35-37]. Three of the studies with CAS THA as study contrast were of high methodological quality [15,16,40], the other four medium methodological quality [18,38,39,41].

**Table 3:** Results of the methodological quality assessment*

	Fulfilled validity criteria				Unfulfilled validity criteria	Incomplete information for validity assessment	Internal validity score	Methodological quality
					
Study	Selection bias (1,2,3)	Performance bias (5)	Attrition bias (6,8)	Detection bias (4,7)				
**MIS**								
Lawlor et al. [31]	1,2,3	5	6	4,7	8	-	7	High
Chimento et al. [4]	1,2,3	5	6	4,7	8	-	7	High
Ogonda et al. [34]	1,2,3	5	6	4,7	8	-	7	High
Kim [30]	1,3	5	6	4,7	2,8	-	6	High
Bennett et al. [22]	3	5	6	4,7	8	1,2	5	Medium
Chung et al. [23]	3	5	6	4,7	1,2,8	-	5	Medium
Khan et al. [29]	3	5	6	4,7	1,2,8	-	5	Medium
Dorr et al. [26]	3	5	6	4,7	8	1,2	5	Medium
Ciminiello et al. [24]	3	5	6	7	1,2,4,8	-	4	Medium
Dutka et al. [27]	3	-	6	4,7	1,2,8	5	4	Medium
Hart et al. [28]	3	-	6	4,7	8	1,2,5	4	Medium
Mazoochian et al. [37]	3	5	-	4,7	8	1,2,6	4	Medium
Rittmeister & Peters [35]	3	5	6	7	1,2,4,8	-	4	Medium
Speranza et al. [36]	3	5	6	7	4,8	1,2	4	Medium
DiGioia et al. [17]	3	5	-	4,7	1,2,8	6	4	Medium
De Beer et al. [25]	3	-	6	7	1,2,4,8	5	3	Medium
Levine et al. [32]	3	-	6	7	1,2,4,5,8	-	3	Medium
Nakamura et al. [33]	3	-	6	7	1,2,4,5,8	-	3	Medium
								
**CAS**								
Leenders et al. [16]	1,2,3	5	6	4,7	8	-	7	High
Parratte & Argenson [40]	1,2,3	5	6	4,7	8	-	7	High
Kalteis et al. [15]	1,3	5	6	4,7	8	2	6	High
Kalteis et al. [38]	1,3	5	6	7	8	2,4	5	Medium
Sugano et al. [41]	3	5	6	4,7	1,2,8	-	5	Medium
Najarian et al. [39]	3	5	6	7	1,2,8	4	4	Medium
Wixson & MacDonald [18]	3	5	6	7	1,2,8	4	4	Medium

* Methodological quality criteria are presented in Table 1.

### Operative time

Operative time was reported in 16 studies with MIS THA as study contrast (Table 4). Two high-quality studies and five medium-quality studies reported a moderate to large decrease in operative time for MIS THA [23,27,30,32-34,37]. One other high-quality study and eight medium-quality studies reported no significant difference in operative time [4,17,24-26,28,29,35,36].

**Table 4:** Results of perioperative outcome measures*

			Operative time	Intraoperative blood loss	Length of Stay
			
Study	Methodological quality	No. of patients	SMD (95% CI)	SMD (95% CI)	SMD (95% CI)
**MIS**					
Chimento et al. [4]	High	60	0.03 (-0.48, 0.54)	-0.74 (-1.26, -0.21)	NE (NS)
Ogonda et al. [34]	High	219	-0.49 (-0.76, -0.22)	-0.29 (-0.56, -0.03)	NE (NS)
Kim [30]	High	140	NE (S, decrease)	NE (NS)	NR
Chung et al. [23]	Medium	120	-0.42 (-0.79, -0.06)	-1.18 (-1.56, -0.79)	-0.73 (-1.10, -0.36)
Khan et al. [29]	Medium	200	-0.01 (-0.29, 0.26)	-0.84 (-1.13. -0.55)	NR
Dorr et al. [26]	Medium	60	-0.32 (-0.83, 0.19)	-0.41 (-0.92, 0.10)	-0.53 (-1.03, -0.03)
Ciminiello et al. [24]	Medium	120	NE (NS)	NE (NS)	NE (NS)
Dutka et al. [27]	Medium	120	-0.88 (-1.25, -0.50)	-1.40 (-1.80, -1.00)	NE (NS)
Hart et al. [28]	Medium	120	NE (NS)	NR	NR
Mazoochian et al. [37]	Medium	52	NE (S, decrease)	NE (S, decrease)	NR
Rittmeister & Peters [35]	Medium	152	NE (NS)	NE (NS)	NR
Speranza et al. [36]	Medium	100	NE (NS)	NE (S, decrease)	NE (NS)
DiGioia et al. [17]	Medium	70	NE (NS)	NR	NE (NS)
De Beer et al. [25]	Medium	60	NE (NS)	-0.77 (-1.30, -0.25)	NE (NS)
Levine et al. [32]	Medium	201	NE (S, decrease)	NE (NS)	NE (S, decrease)
Nakamura et al. [33]	Medium	92	-0.85 (-1.28, -0.42)	-0.42 (-0.84, -0.01)	NR
					
**CAS**					
Kalteis et al. [15] (CT-based)	High	60	NE (S, increase)	NR	NR
Kalteis et al. [15] (Imageless)	High	60	NE (NS)	NR	NR
Kalteis et al. [38]	Medium	45	0.45 (-0.14, 1.04)	NR	NR
Sugano et al. [41]	Medium	180	NE (S, increase)	NE (NS)	NR
Najarian et al. [39]	Medium	100	NE (S, increase)	NE (S, decrease)	NR

SMD = standardized mean difference; 95% CI = 95% confidence interval; NE = SMD not estimable; S = reported differences between groups were significant; NS = reported differences between groups were not significant; NR = outcome measure not reported.

* A negative SMD with 95% CI indicates a decrease in operative time, intraoperative blood loss and length of stay in favor of the study group.

Operative time was reported in four studies with CAS THA as study contrast (Table 4). Except for the sub-study of Kalteis et al. [15] on an imageless computer navigation system, all studies reported a moderate increase in operative time for THA when using computer navigation [15,38,39,41].

### Intraoperative blood loss

Intraoperative blood loss was reported in 14 studies with MIS THA as study contrast (Table 4). Two high-quality studies [4,34] and eight medium-quality studies [23,25-27,29,33,36,37] reported a small-to-large decrease in intraoperative blood loss after MIS THA. One high-quality study and three medium-quality studies reported no significant difference [24,30,32,35].

Two studies with CAS THA as study contrast reported on intraoperative blood loss (Table 4). Sugano et al. [41] reported no significant effect of the use of computer navigation during THA on intraoperative blood loss. However Najarian et al. [39] reported a significant decrease in intraoperative blood loss.

### Length of stay

Ten studies reported on length of stay after MIS THA (Table 4). Three medium-quality studies reported a moderate-to-large decrease in length of hospital stay after MIS THA [23,26,32]. Two high-quality studies [4,34] and five medium-quality studies [17,24,25,27,36] reported no significant differences in length of stay between the MIS THA group and the control group. None of the studies with CAS THA as study contrast reported data on length of stay.

### Complications

Seventeen studies with MIS THA as study contrast reported on intraoperative and postoperative complications (Table 5). Two high-quality studies [4,30] and two medium-quality studies [35,37] reported higher complication rates after MIS THA, but these rates were statistically non-significant. The results of six medium-quality studies [23,25,26,29,32,33] showed lower, though statistically non-significant, complication rates after MIS THA. Moreover, two high-quality studies [31,34] (reporting on the same data) and five medium-quality studies [17,24,27,28,35] reported no differences in complication rates between the study and control group.

**Table 5:** Operative complications and acetabular outliers*

		No. of complications			No. of outliers		
		
Study	Methodological quality	Study group	Control group	OR (95% CI)	Study group	Control group	OR (95% CI)
**MIS**							
Lawlor et al. [31]^†^	High	3/109	4/110	0.75 (0.16, 3.43)			NR
Chimento et al. [4]	High	3/28	2/32	1.80 (0.28, 11.64)	0	0	-
Ogonda et al. [34]^†^	High	3/109	6/110	0.75 (0.16, 3.43)	16/105	19/109	0.85 (0.41, 1.76)
Kim [30]	High	3/70	2/70	1.52 (0.25, 9.40)	13/70	11/70	1.22 (0.51, 2.95)
Chung et al. [23]	Medium	3/57	5/55	0.58 (0.13, 2.54)	0	0	-
Khan et al. [29]	Medium	15/100	21/100	0.66 (0.32, 1.38)	3/100	3/100	1.00 (0.20, 5.08)
Dorr et al. [26]	Medium	2/30	3/30	0.64 (0.10, 4.15)	0	0	-
Ciminiello et al. [24]	Medium	0	0	-	0	0	-
Dutka et al. [27]	Medium	1/60	1/60	1.00 (0.06, 16.37)	0	0	-
Hart et al. [28]	Medium	1/60	1/60	1.00 (0.06, 16.37)	0	0	-
Mazoochian et al. [37]	Medium	4/26	3/26	1.39 (0.28, 6.95)			NR
Rittmeister & Peters [35]	Medium	7/76	6/76	1.18 (0.38, 3.70)			NR
Speranza et al. [36]	Medium	3/46	0/54	8.77 (0.44, 174.38)	1/46	3/54	0.38 (0.04, 3.76)
DiGioia et al. [17]	Medium	0	0	-	0	0	-
De Beer et al. [25]	Medium	1/30	2/30	0.48 (0.04, 5.63)	0	0	-
Levine et al. [32]	Medium	14/126	13/75	0.60 (0.26, 1.35)			NR
Nakamura et al. [33]	Medium	1/50	2/42	0.41 (0.04, 4.67)	4/50	5/42	0.64 (0.16, 2.57)
							
**CAS**							
Leenders et al. [16]	High			NR	7/50	14/50	0.42 (0.15, 1.15)
Parratte & Argenson [40]	High	0	0	-	6/30	17/30	0.19 (0.06, 0.60)
Kalteis et al. [15] (CT-based)	High	0/30	1/30	0.32 (0.01, 8.24)	5/30	16/30	0.17 (0.05, 0.58)
Kalteis et al. [15] (Imageless)	High	0/30	1/30	0.32 (0.01, 8.24)	2/30	16/30	0.06 (0.01, 0.31)
Kalteis et al. [38]	Medium	0	0	-	2/23	11/22	0.10 (0.02, 0.51)
Sugano et al. [41]	Medium	0/60	7/120	0.13 (0.01, 2.23)	0/59	31/111	0.02 (0.00, 0.36)
Najarian et al. [39]	Medium	2/47	2/53	1.13 (0.15, 8.38)	6/47	18/53	0.28 (0.10, 0.80)
Wixson & MacDonald [18]	Medium	2/82	1/50	1.23 (0.11, 13.87)	17/82	18/50	0.46 (0.21, 1.02)

No. = number; OR = Odds ratio; 95% CI = 95% confidence interval; NR = outcome measure not reported. ^† ^Articles report on the same study population.

* An OR below 1 with 95% CI indicates lower odds for complications and outliers in favor of the study group.

Seven studies with CAS THA as study contrast reported on intraoperative and postoperative complications (Table 5). Both sub-studies of Kalteis et al. [15], which are high-quality studies, reported lower complication rates in the CAS group than in the control group. These results are also shown in a medium-quality study [41], yet in all these studies such differences in complication rates were statistically non-significant. One high-quality study [40] and three medium-quality studies [18,38,39] reported no significant difference either.

### Acetabular outliers

The number of acetabular components outside the desired alignment range (acetabular outliers) was reported in 13 studies with MIS THA as study contrast (Table 5). The high-quality study of Kim [30] reported more acetabular outliers in the study group, but these rates were statistically non-significant. Fewer acetabular outliers were reported in one high-quality study [34] and two medium-quality studies [33,36], though this difference was also non-significant. In addition, one high-quality study [4] and eight medium-quality studies [17,23-29] reported no differences in acetabular outliers.

All studies with CAS THA as study contrast reported on the number of acetabular outliers (Table 5). Five studies showed significant fewer acetabular outliers for CAS THA [15,38-41]. The other two studies also reported fewer acetabular outliers for CAS THA, but this difference was statistically non-significant [16,18].

### Physical functioning

In order to evaluate physical functioning after THA, several physician-based and self-reported questionnaires are in use. Furthermore, objective assessment of physical function can be done by means of gait analysis. In total, thirteen studies with MIS THA as study contrast reported on physical functioning outcome measures. None of the studies with CAS as study contrast assessed physical functioning of patients after THA.

#### Physician-reported physical functioning

Ten studies with MIS THA as study contrast reported on physician-based physical functioning outcome measures (Table 6). In these studies, two different outcome measures were used, namely the Harris Hip Score [17,24-27,34,36,37] and the Merle d'Aubigné Hip Score [28,33]. Six studies reported six weeks postoperatively follow-up data. One medium-quality study [27] reported significant improvements in physician-reported physical functioning, and the other five studies (one high-quality and four medium-quality) reported no significant differences [24-26,34,37]. Five studies reported three months postoperatively follow-up data. Three medium-quality studies [17,28,37] reported significant improvement in physical functioning scores in favour of MIS THA, and two medium-quality studies [27,36] showed no significant differences. Six medium-quality studies reported six months postoperatively follow-up data. Only one study [17] reported significant improvement in physical functioning scores six months after MIS THA when compared to conventional THA; the other five studies [26-28,33,36] showed no significant differences. Two medium-quality studies reported follow-up data at one year after THA [17,28]. Neither study found significant differences in physical function.

**Table 6:** Results of outcome measures to evaluate physical functioning after MIS THA*

		Follow-up			
		
Study	Methodological quality	6 weeks	3 months	6 months	1 year
**Physician-reported**					
Ogonda et al. [34] *	High	0.08 (-0.18, 0.35)	NR	NR	NR
Dorr et al. [26] *	Medium	NE (NS)	NR	NE (NS)	NR
Ciminiello et al. [24] *	Medium	0.26 (-0.10, 0.62)	NR	NR	NR
Dutka et al. [27] *	Medium	NE (S) ^a^	NE (NS)	NE (NS)	NR
Speranza et al. [36] *	Medium	NR	NE (NS)	NE (NS)	NR
Hart et al. [28]^†^	Medium	NR	NE (S) ^a^	NE (NS)	NE (NS)
Mazoochian et al. [37] *	Medium	NE (NS)	NE (S) ^a^	NR	NR
DiGioia et al. [17] *	Medium	NR	NE (S) ^a^	NE (S) ^a^	NE (NS) ^a^
De Beer et al. [25] *	Medium	0.40 (-0.11, 0.91)	NR	NR	NR
Nakamura et al. [33]^†^	Medium	NR	NR	NE (NS)	NR
					
**Patient-reported**					
Ogonda et al. [34]^‡^	High	0.03 (-0.23, 0.30)	NR	NR	NR
Ogonda et al. [34]^§^	High	0.13 (-0.13, 0.40)	NR	NR	NR
Ogonda et al. [34] **	High	0.01 (-0.26, 0.27)	NR	NR	NR
Khan et al. [29]^‡^	Medium	NR	NE (S) ^a^	NR	NE (S) ^a^
Khan et al. [29]^††^	Medium	NR	NE (NS)	NR	NR
Mazoochian et al. [37]^‡^	Medium	NE (S) ^a^	NE (S) ^a^	NR	NR
Speranza et al. [36]^‡^	Medium	NR	NE (NS)	NE (NS)	NR
De Beer et al. [25]^§^	Medium	0.24 (-0.27, 0.74)	NR	NR	NR
					
**Gait analysis ^b^**					
Lawlor et al. [31]	High	-0.10 (-0.37, 0.16)	NR	NR	NR
Ogonda et al. [34]	High	0.19 (-0.07, 0.46)	NR	NR	NR
Bennet et al. [22]	Medium	NE (NS)	NR	NR	NR
Dorr et al. [26]	Medium	NE (NS)	NE (NS)	NR	NR

Scores are reported as SMD (95%CI). NE = SMD not estimable; S = reported differences between groups were significant; NS = reported differences between groups were not significant; NR = outcome measure not reported. * scores on the HHS; ^† ^scores on the Merle d'Aubigné Hip score; ^‡ ^scores on the WOMAC; ^§ ^scores on the OHS; ** scores on the physical component of the SF-36; ^†† ^scores on the physical component of the SF-12. ^a ^Improvement in score. ^b ^Gait velocity is used as outcome measure for gait analysis.

* A positive SMD with 95% CI indicates better physical functioning in favor of the study group.

#### Patient-reported physical functioning

Five studies with MIS THA as study contrast reported on patient-reported physical functioning by means of two disease-specific outcome measures, namely the Western Ontario McMaster University Osteoarthritis Index (WOMAC) [29,34,36,37] the Oxford Hip Score (OHS) [25,34] (Table 6). Two of these studies also reported on the physical component of the MOS 36-item Short Form Health Survey (SF-36) [29] and the Short Form-12 (SF-12) [34], which are both generic questionnaires to assess health-related quality of life. Three studies reported six weeks postoperatively follow-up data. One high-quality study [34] and one medium-quality study [25] reported no to small but non-significant improvements on patient-reported physical function. However, one medium-quality study [37] reported significant effects on the WOMAC in favour of MIS THA. Three medium-quality studies reported follow-up data of three months after MIS THA [29,36,37]. Two of these studies reported significant effects on the WOMAC in favour of MIS THA [29,37] and no significant difference on the physical component scale of the SF-12 [29]. Speranza et al. [36] showed no difference on the WOMAC. One medium-quality study [36] reported no significant differences on the WOMAC six months after MIS THA. Another medium-quality study [29] however reported significant differences on the WOMAC one year postoperatively.

#### Gait analysis

Four studies with MIS THA as study contrast reported gait analysis data to evaluate physical function after THA (Table 6). All four studies reported six weeks postoperatively follow-up data. Two high-quality studies [31,34] and two medium-quality studies [22,26] reported no significant effect on gait function. Only one medium-quality study [26] reported on three months postoperatively follow-up data. They reported no significant effect on gait function three months after MIS THA. Furthermore, none of the studies reported on follow-up data of six months and one year postoperatively.

### Pain

Five studies with MIS THA as study contrast reported on pain (Table 7). One study was of high quality [30], the other four studies of medium quality [17,27,28,33]. These studies used three different measures to assess pain: a Visual Analogue Scale (VAS) [27,30] for pain, the subscale of the Merle d'Aubigné Hip score [28,33], and the pain subscale of the Harris Hip Score [17]. Three studies reported six weeks postoperatively follow-up data, reporting a significant moderate decrease [27] and no significant effect [28,30] of MIS THA on pain. No significant differences in pain were reported at three months [17,27,30], six months [17,27,28,33] and one year [17,28,30] postoperatively. None of the studies with CAS as study contrast reported on pain after THA.

**Table 7:** Results of outcome measures to evaluate pain after THA.

		Follow-up			
		
Study	Methodological quality	6 weeks	3 months	6 months	1 year
Kim[30]*	High	NE (NS)	NE (NS)	NR	NE (NS)
Dutka et al. [27] *	Medium	-0.51 (-0.87, -0.15)	-0.13 (-0.49, 0.23)	-0.31 (-0.67, 0.05)	NR
Hart et al. [28]^†^	Medium	NE (NS)	NR	NE (NS)	NE (NS)
DiGioia et al. [17]^‡^	Medium	NR	NE (NS)	NE (NS)	NE (NS)
Nakamura et al. [33]^†^	Medium	NR	NR	NE (NS)	NR

Scores are reported as SMD (95%CI). NE = SMD not estimable; S = reported differences between groups were significant; NS = reported differences between groups were not significant; NR = outcome measure not reported. * score on a VAS for pain; ^† ^score on the pain subscale of the Merle d'Aubigné Hip score; ^‡ ^score on the pain subscale of the HHS.

### Best-evidence synthesis

#### MIS THA

Compared to conventional THA, strong evidence was found for a decrease in operative time and operative blood loss after MIS THA. The evidence for a shorter length of stay was moderate. Strong evidence was also found for no difference in complication rates and position of the acetabular component. Moderate to strong evidence was found for no difference in physical functioning six weeks and six months after surgery. The evidence of a positive effect of MIS THA on physical functioning three months postoperatively was conflicting, as was the evidence for less pain after MIS THA six weeks postoperatively. The evidence for no differences in pain levels three and six months after surgery was strong.

#### CAS THA

Strong evidence was found for a positive effect of CAS THA on the position of the acetabular component. The evidence for a positive effect on operative blood loss was limited. Strong evidence was found for an increase in operative time and for no significant difference in complication rates after CAS THA.

## Discussion

We have reviewed the current literature evaluating the effectiveness of MIS, CAS and computer-assisted MIS for THA. The extensive literature search resulted in 18 articles with MIS THA as study contrast, and seven with CAS THA as study contrast, yet no study with computer-assisted MIS for THA as study contrast was discovered. The results of this systematic review indicate that there were no significant differences in operative complications and acetabular component positioning between MIS THA and the conventional procedure. Furthermore, MIS THA resulted in a reduction in blood loss, operative time and reduced length of stay. The added value of MIS THA over the conventional procedure in terms of a faster functional recovery however remains to be proven. Computer-assisted THA results in better positioning of the acetabular component. It may also have a positive effect on operative blood loss and complications despite an increased operative time.

Contrary to what proponents of MIS THA stated, this review showed that MIS THA had no effect on physical functioning, as measured by questionnaires as well as gait analysis. Since the main purported benefit of MIS THA is a decrease in the amount of soft-tissue (muscle) damage, it can be postulated that a difference in improvement of physical functioning and pain will only be seen in the early postoperative period. Only eight studies reported data on physical functioning at six weeks postoperatively [22,24-27,31,34,37]. Six of these studies assessed physical functioning by means of either surgeon-reported or patient-reported questionnaires [24-27,34,37]. Although these are shown to be useful for detecting changes in physical functioning over time in patients with osteoarthritis of the hip and after THA [42,43], it is arguable whether these questionnaires are sensitive enough to detect subtle differences in improvement of physical functioning after conventional or MIS THA. A possible solution for this problem is to measure physical functioning objectively by means of quantitative gait analysis. However, only four studies assessed physical functioning using gait analysis [22,26,31,34]. Quantitative gait analysis has been used for numerous applications and has provided insights into functional characteristics not identifiable by clinical exam or other methods. Several studies have compared two surgical techniques for THA, attempting to identify differences in functional outcome [44-46]. The studies that used gait analysis [44,46] revealed differences between the surgical approaches, while this result failed to be identified by means of a questionnaire [45].

The results for CAS THA demonstrate an increase in operative time and limited evidence for a decrease in operative blood loss, but CAS THA had no effect on operative complication rates. Additionally, the use of CAS during THA had a positive effect on the outliers of the acetabular component position outside the desired range. These results justify use of computer navigation during THA. With improved surgery patients should benefit from having lower morbidity rates, better functional outcome and greater longevity of implants [12]. Wines and McNicol [47] showed that during conventional THA it is technically difficult to achieve an accurate alignment of the acetabular component intraoperatively. As judged by postoperative CT scans, surgeons' intraoperative estimates of acetabular component positioning were inside the desired range in less than two-thirds of the cases. Since accurate component positioning benefits the longevity of the implanted prosthesis, CAS can help achieve this goal. However, broader application of computer navigation systems is still hindered by increased operative times, partly due to the complexity of the systems and the accompanying financial costs.

Despite efforts to get an ample overview of the available literature on MIS and CAS for THA, no articles with computer-assisted MIS for THA as study contrast were discovered. Some of the studies included compared computer-assisted MIS for THA with either MIS THA [18,39] or CAS THA [17,26]. Their results are in line with the other studies included in this review that compared MIS THA or CAS THA with a conventional approach. Still, an additive effect of the combination of MIS and CAS for THA needs to be established.

Some critical remarks can be made on the included studies. First, a wide variety of surgical approaches was used in them. We chose to analyse all surgical approaches together, since the aim of this systematic review was to assess the effectiveness of minimally invasive THA, but not of any specific minimally invasive THA approach. Second, the surgical approaches were too heterogeneous and often poorly described to perform subgroup analyses. Studies on image-based and imageless navigation systems were also analysed together, since research has shown that imageless navigation is as reliable as image-based navigation for positioning the acetabular component [15]. Third, the studies included in this review use a variety of definitions of 'minimally invasive THA' or 'mini-incision THA'. The term 'minimally invasive' is clearly open to interpretation. There are patent differences between using an alternate surgical approach intended to gain access to the hip joint through less soft-tissue dissection and using intermuscular planes, and performing the conventional procedure through a smaller skin incision. In the literature, studies use the term 'mini-incision' while, according to the description of the surgical technique, it is a minimally invasive technique which has been used. Conversely, the term 'minimally invasive' is also used in the literature to indicate what appears to be a mini-incision technique. Fourth, the used definitions for the desired range of acetabular component angle varied enormously in the published results of MIS THA and CAS THA. The majority of the studies use the safe zone recommended by Lewinnek et al [10], including an abduction angle of 40 ± 10° and an anteversion angle of 15 ± 10°. Some studies reported slightly different operation goals, depending on the surgical approach used. The operation goal was nonetheless always the same in the study group and the control group. Finally, not all studies reported the experience of the surgeons with the specific surgical technique. The introduction of a new surgical technique is often accompanied by a learning curve, associated with a temporary increase of adverse events [48]. To make an objective comparison between conventional technique and a minimally invasive or computer-assisted technique for THA, it is crucial to exclude the cases that are operated on during the time span of the learning curve for the new surgical technique.

Some limitations of this review and its conclusions need to be addressed. In this systematic review, a highly sensitive comprehensive search was conducted following the recommendations of the Cochrane collaboration in order to identify articles of interest. For practical reasons though, only studies published in English, Dutch or German were included in the final review, which might have led to selection bias. Additionally, in order to get a broad overview of all the literature on MIS, CAS and computer-assisted MIS for THA, we chose to include not only RCTs but also CCTs. Shrier et al. [49] stated that including studies other than RCTs may provide important additional information, thereby improving inference of the results. Moreover, Poolman et al. [50] suggested that readers should not assume that studies labelled as Level I are of a high reporting quality, or of a better reporting quality than Level II studies. This was also seen in the present review; some CCTs were of a higher methodological quality than several of the included RCTs. Of the studies included, only six were considered of high quality. None of the studies conducted their analyses following the intention-to-treat principle. Furthermore, several RCTs failed to report on the methods of randomisation and treatment allocation. Since several studies failed to report sufficient data to calculate SMDs, it was not possible to conduct a meta-analysis (quantitative statistics). We therefore used qualitative levels of evidence to summarize the results. Use of a best-evidence synthesis is a next best solution and is a transparent method commonly applied when statistical pooling is not feasible or clinically viable [20].

## Conclusions

The results of this systematic review indicate that MIS THA is a safe surgical procedure, without increases in operative time, blood loss, operative complications and component positioning when compared to the conventional procedure. However, the surplus value of MIS THA over the conventional procedure in terms of a faster functional recovery remains to be proven. The results of this review also indicate that computer-assisted THA, despite an increased operative time, may have a positive effect on operative blood loss and complications. More importantly, the use of CAS during THA results in better positioning of the acetabular component of the prosthesis. Since minimally invasive THA and the use of computer navigation are becoming increasingly popular in orthopaedics, combining 'the best of both worlds' would be a sensible next step to take. With respect to future research, well-designed studies on MIS THA, CAS THA and especially computer-assisted MIS THA are needed, in which the used definitions, surgical technique, study population, outcome measures and study end-points are adequately described.

## Competing interests

The authors declare that they have no competing interests.

## Authors' contributions

IHFR co-coordinated the review, contributed to the literature search, and performed the data extraction, statistical analyses and drafting of the manuscript. BPH contributed to the literature search, data extraction and drafting of the manuscript. MS and WZ participated in the study design and have been involved in, together with SKB, JWG, ALB and RW, critically revising the manuscript. All authors read and approved of the final manuscript.

## Pre-publication history

The pre-publication history for this paper can be accessed here:

http://www.biomedcentral.com/1471-2474/11/92/prepub

## Supplementary Material

Additional file 1 **Study characteristics**. Characteristics of the included studies. Click here for file
