# Supplementary material for: Minimally invasive and computer-navigated total hip arthroplasty: a qualitative and systematic review of the literature
Source: BMC Musculoskelet Disord. 2010 May 17;11:92. doi: 10.1186/1471-2474-11-92 (PMC2879237; doi:10.1186/1471-2474-11-92)
Supplement: Additional file 1 — Study characteristics. Characteristics of the included studies. [file 1471-2474-11-92-S1.PDF]

Additional file 1. Study characteristics*

Study	N	Method	Participants	Interventions	Outcome measures	Follow-up
**MIS**						
Bennett et al. [22]	17	RCT	Chosen at random from a larger study cohort of 200 patients who were on a waiting list for THA. Exclusion criteria: previous surgery to ipsilateral hip, polyarthritis.	S: minimally invasive posterior approach (<10 cm)<br>C: standard posterior approach (16 cm)	Gait velocity.	6 weeks
Chimento et al. [4]	60	RCT	Patients undergoing THA between Nov. 15, 1999 and July 15, 2000. Exclusion criteria: BMI >30, and a hip pathology that would require a more extensile exposure to reconstruct the hip.	S: minimally invasive posterolateral approach (± 8 cm)<br>C: standard posterolateral approach (± 15 cm)	Operative time; intraoperative blood loss; length of hospital stay; complications; radiographic evaluation.	
Chung et al. [23]	120	CCT	Patients with OA. Exclusion criteria: weight >100kg, semi-ankylosed joints, severe protrusio or dysplasia. Matched for age, weight and diagnosis.	S: mini-incision posterolateral approach<br>C: standard posterior approach	Operative time; intraoperative blood loss; length of hospital stay; complications; radiographic evaluation.	
Ciminiello et al. [24]	120	CCT	Patients with OA, assigned by a surgeon to one of the two groups. Matched for age, gender, BMI, ASA score, diagnosis, prosthesis, type of fixation, anesthesia, surgical approach and intraoperative patient positioning.	S: small incision (<5 inches)<br>C: conventional incision (≥5 inches)	Operative time, intraoperative blood loss; length of hospital stay; complications; radiographic evaluation; HHS.	6 weeks
De Beer et al. [25]	60	CCT	Patients undergoing primary unilateral THA. Matched for gender, age, BMI, preoperative diagnosis of osteoarthritis and ASA score.	S: minimally invasive direct lateral approach (≤10 cm)<br>C: standard direct lateral approach	Operative time; intraoperative blood loss; length of hospital stay; complications; radiographic evaluation; HHS, OHS.	6 weeks
DiGioia et al. [17]	70	CCT	Patients with osteoarthritis. Matched for gender, age, and diagnosis.	S: minimally invasive posterior approach with computer-assisted navigation (CT-based)<br>C: standard posterior approach with computer-assisted navigation (CT-based)	Length of hospital stay; HHS.	3,6, and 12 months
Dorr et al. [26]	60	RCT	Patients undergoing primary unilateral THA between Jan. 2004 and Oct. 2005. Exclusion criteria: previous surgery on the affected hip, a pathological condition of the hip that required an extensile exposure, same-day bilateral THA and inflammatory polyarthritis.	S: mini-incision posterior approach with computer –assisted navigation (Imageless) (10 ± 2 cm)<br>C: standard posterior approach with computer –assisted navigation (Imageless) (20 ± 2 cm)	Operative time; intraoperative blood loss; length of hospital stay; complications; radiographic evaluation; HHS.	6 weeks, 3 and 6 months
Dutka et al. [27]	120	RCT	Patients undergoing THA.	S: minimally invasive direct lateral approach (6-8 cm)<br>C: standard direct lateral approach (20-25 cm)	Operative time; intraoperative blood loss; length of hospital stay; complications; radiographic evaluation; HHS; VAS pain.	6 weeks, 3 and 6 months

Hart et al. [28]	120	RCT	Patients undergoing THA between Sept. 2000 and Feb. 2002, with an age >65 years and a BMI<35. Exclusion criteria: patients affected by coagulation disorders and an Hb < 12 g/dl.	S: minimally invasive posterolateral approach (9-10 cm)<br>C: standard posterolateral approach (20 cm)	Operative time; complications; radiographic evaluation; Merle d'Aubigné-Charnley score.	6 weeks, 6 and 12 months
Khan et al. [29]	200	CCT	Patients with osteoarthritis or rheumatoid arthritis, undergoing THA. Exclusion criteria: revision arthroplasty, congenital hip anomaly, previous hip surgery or infection and iatrogenic damage to the piriformis. Matched for age, gender, ASA, BMI and primary diagnosis. Retrospective control group.	S: less-invasive posterior approach<br>C: standard posterior approach	Operative time; intraoperative blood loss; complications; radiographic evaluation, WOMAC, SF-12.	6 weeks, 3 and 12 months
Kim [30]	140	RCT	Patients undergoing bilateral THA; 1 minimally invasive and 1 standard THA.	S: minimally invasive modified posterolateral approach (8 cm)<br>C: standard posterolateral approach (15-20 cm)	Operative time; intraoperative blood loss; complications; radiographic evaluation.	6 weeks, 3, 6 and 12 months
Lawlor et al. [31]	210	RCT	Patients undergoing THA. Exclusion criteria: previous surgery to affected hip and inflammatory polyarthritis if the severity of the disease was likely to compromise postoperative mobility.	S: minimally invasive posterior approach (≤ 10 cm)<br>C: standard posterior approach (16 cm)	Gait velocity.	6 weeks
Levine et al. [32]	201	CCT	Primary THA. Inclusion criteria for MIS: BMI<35, adequate home support and motivation for accelerated rehabilitation, no significant deformity. Standard operation: all other patients.<br>Retrospective control group.	S: minimally invasive two-incision anterior approach (one incision of 5cm and one of 2-3 cm)<br>C: modified Hardinge approach (10-15 cm)	Operative time; intraoperative blood loss; length of hospital stay; complications.	
Mazoochian et al. 37]	52	RCT	Patients with indication for a cementless THA. Exclusion criteria: patients in which an acetabular plastic had to be performed or patients with a malignancy.	S: modified Hardinge approach (8 cm)<br>C: standard lateral approach by Bauer	Operative time; intraoperative blood loss; complications; HHS; WOMAC.	6 weeks and 3 months
Nakamura et al. [33]	92	CCT	Patients with osteoarthritis or avascular necrosis. Retrospective control group.	S: mini-incision posterior approach<br>C: standard posterior approach (15-20 cm)	Operative time; intraoperative blood loss; radiographic evaluation.	
Ogonda et al. [34]	219	RCT	Patients undergoing THA. Exclusion criteria: history of previous surgery on the affected hip and inflammatory polyarthritis if the severity of the disease was likely to compromise postoperative mobility.	S: mini-incision posterior approach<br>C: standard posterior approach	Operative time; intraoperative blood loss; length of hospital stay; complications; radiographic evaluation; HHS; OHS; WOMAC; SF-36.	6 weeks
Rittmeister & Peters [35]	152	CCT	Patients undergoing THA.<br>Retrospective control group.	S: minimally invasive posterior approach<br>C: standard anterolateral approach	Operative time; intraoperative blood loss; complications.	
Speranza et al. [36]	100	RCT	Patients undergoing THA.	S: mini-incision lateral approach<br>C: standard lateral approach	Operative time; intraoperative blood loss; length of hospital stay; complications; radiographic evaluation; HHS; WOMAC.	3 and 6 months

**CAS**					
Kalteis et al. [38]	45	RCT	Patients with primary osteoarthritis undergoing THA.	S: standard anterolateral approach with computer-assisted navigation (CT-based)<br>C: standard anterolateral approach	Operative time; complications; radiographic evaluation.
Kalteis et al. [15]	90	RCT	Patients with osteoarthritis undergoing THA. Exclusion criteria: arthritis secondary to hip dysplasia, post-traumatic deformities of the pelvis, or age <50 years.	S1: modified transgluteal approach with computer-assisted navigation (CT-based)<br>S2: modified transgluteal approach with computer-assisted navigation (Imageless)<br>C: modified transgluteal approach	Operative time; radiographic evaluation.
Leenders et al. [16]	100	RCT	Patients with osteoarthritis undergoing THA.	S: anterolateral approach with computer-assisted navigation (CT-based)<br>C: standard anterolateral approach	Radiographic evaluation.
Najarian et al. [39]	100	CCT	Patients undergoing THA. Retrospective control group.	S: minimally invasive single-incision posterior approach with computer-assisted navigation (Imageless)<br>C: minimally invasive single-incision posterior approach	Operative time; intraoperative blood loss; complications; radiographic evaluation.
Parratte & Argenson [40]	60	RCT	Patients undergoing THA. Inclusion criteria: age 20-80 years and weight <100kg. Exclusion criteria: trochanteric osteotomy or revision hip surgery. Matched for gender, age, pathological condition, operatively treated side and BMI.	S: anterolateral approach with computer-assisted navigation (Imageless)<br>C: anterolateral approach	Radiographic evaluation.
Sugano et al. [41]	180	CCT	Patients undergoing THA.	S: posterolateral approach with computer-assisted navigation (CT-based)<br>C: posterolateral approach	Operative time; intraoperative blood loss; radiographic evaluation.
Wixson & MacDonald [18]	132	CCT	Patients undergoing THA. Retrospective control group.	S: limited posterior approach with computer-assisted navigation (Imageless)<br>C: limited posterior approach	Radiographic evaluation.

* RCT, randomized controlled trial; CCT, controlled clinical trial; S, study group; C, control group; HHS, Harris Hip Score; OHS, Oxford Hip Score; VAS, visual Analog scale; WOMAC, Western Ontario and McMaster Universities Osteoarthritis Index; SF-36, MOS 36-item Short Form Health Survey; SF-12, Short Form-12.
